# A Successful Medical Treatment of Necrotizing Pneumonia in a Pediatric Patient

**DOI:** 10.1155/2020/8875119

**Published:** 2020-12-18

**Authors:** Herlina Uinarni, Felicia Nike, Andi Dwi Bahagia

**Affiliations:** ^1^Department of Anatomy, School of Medicine and Health Sciences, Atma Jaya Catholic University of Indonesia, Jakarta, Indonesia; ^2^Postgraduate Program, Faculty of Medicine, Hasanuddin University, Makassar, South Sulawesi, Indonesia; ^3^Department of Radiology, Grand Family Maternity and Pediatric Hospital, Jakarta, Indonesia; ^4^Department of Pediatrics, Faculty of Medicine, Hasanuddin University, Makassar, South Sulawesi, Indonesia

## Abstract

Necrotizing pneumonia is a rare, serious complication of pneumonia in children. We present a case of a 20-month-old girl presenting with respiratory distress which later be diagnosed with necrotizing pneumonia. In this paper, we highlight the role of imaging such as chest X-ray, chest CT, and lung ultrasonography for diagnosis and the importance of intravenous antibiotic therapy for better outcome.

## 1. Introduction

Necrotizing pneumonia (NP) is a serious complication of pneumonia characterized by progressive illness in a previously healthy child despite treated with antibiotics. The most common pathogens associated with NP in children are *Pneumococci* and *S. aureus* [1]. Managing patients with NP is challenging because there is no firm guidelines outlining management of NP. The mainstay of treatment is supportive care with appropriate antibiotics. Surgical intervention may play a role when medical therapy fails [2].

## 2. Case Presentation

A 20-month-old girl presented to the Emergency Department (ED) with progressive respiratory distress and fever since 1 day before admission. Her weight was 14 kg, her body temperature was 40^o^C, and she had rapid breathing without stridor or cyanosis. She had already been treated by general practitioner 5 days ago with complaints of fever, cough, and difficulty in breathing and had received antipyretic and antiviral drugs with no significant response. Her mother denied history of febrile seizures, stating that she was previously healthy and had no remarkable history of the illness before. She had never received *Streptococcus pneumoniae* and *Haemophilus influenzae* Type b (Hib) vaccine.

Blood sample was obtained for analysis while the patient was in the emergency department and included a complete blood cell count and basic electrolytes. Laboratory values showed a total white blood cell count of 10.100 *μ*l^−1^ constituted of 50% segmented neutrophils. Her hemoglobin was 10.2 g/dL, platelet count was 470.000 *μ*l^−1^, and the C-reactive protein (CRP) was 24 mg/l. The remaining laboratory values were unremarkable.

**Figure 1:**
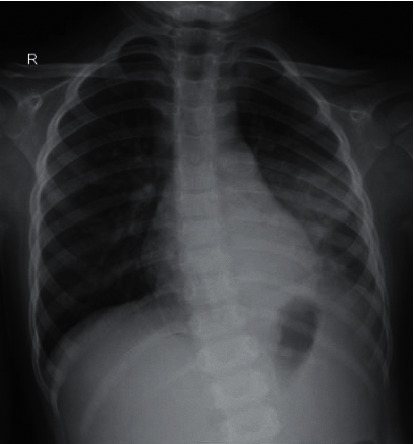
AP-supine chest radiograph: visible consolidation in the left inferior lobe of the lungs. The right lung had no apparent abnormality.

Anteroposterior (AP) view of chest X-ray (CXR) obtained in the radiology department showed left inferior lobar pneumonia (Figure 1). After being hospitalized for 5 days, clinical and laboratory improvements were significant, so her parents asked for discharge upon their own request.

**Figure 2:**
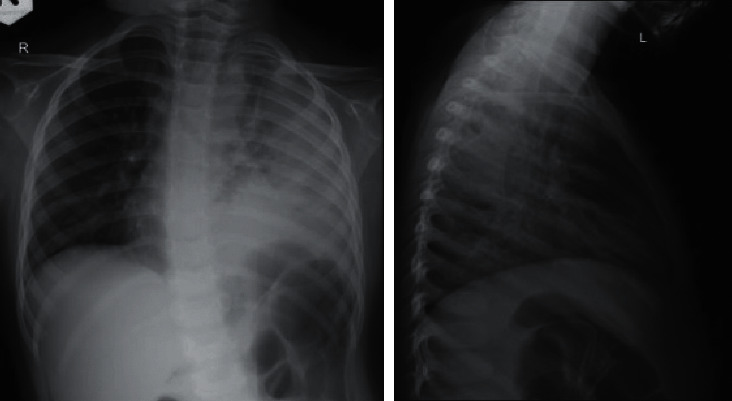
CXR AP and lateral view demonstrating focal area of consolidation in the left inferior lobe dominant. On the AP view, there are multiple small lucency in the left lung field suspicious for possible congenital pulmonary airway malformation (CPAM) and possible pneumonia with air cavitation. Compared to the previous CXR (Figure 1), no lucency was observed.

**Figure 3:**
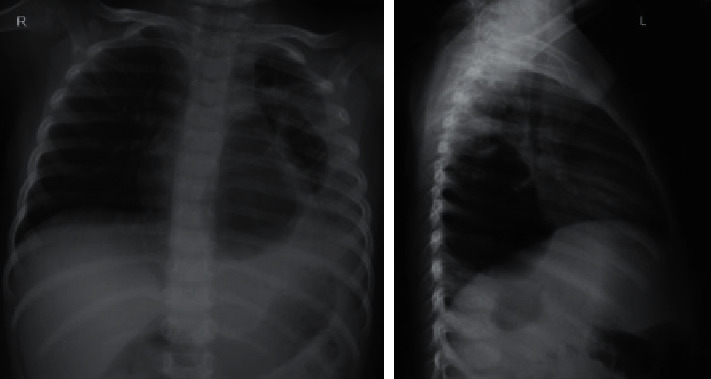
CXR AP and lateral view showing an air-filled cavitary lesion in the infero-posterior left lobe.

**Figure 4:**
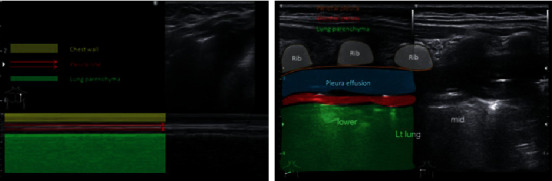
LUS left anterior using M-mode showing no pneumothorax and posterior lower and middle using B-mode: pleural effusion (transudate), pleural irregular thickening, and heterogeneous parenchymal echo texture.

**Figure 5:**
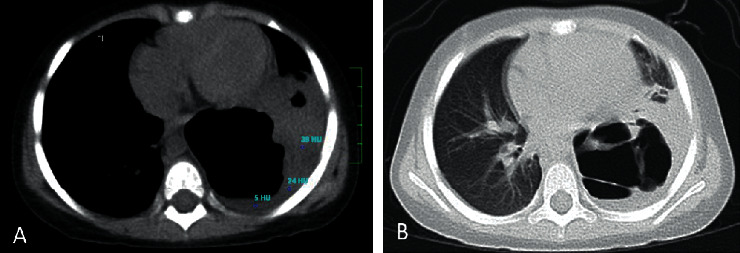
Noncontrast axial-chest CT: mediastinal window (a) and lung window (b). Left pleural effusion density was <25 HU. There was a poorly visible portion of the left lung, and there was air-filled thin wall cavity. Without IV contrast, cavitary necrosis could not be diagnosed because there was no enhancement of the surroundings.

**Figure 6:**
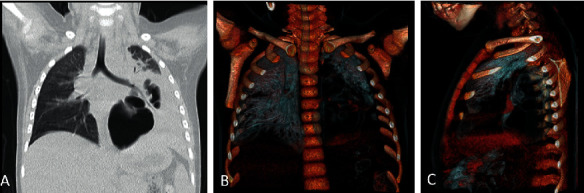
Noncontrast frontal view chest CT: lung window (a) and virtual bronchography (volume-rendered display) for visualization of airways (b, c).

**Figure 7:**
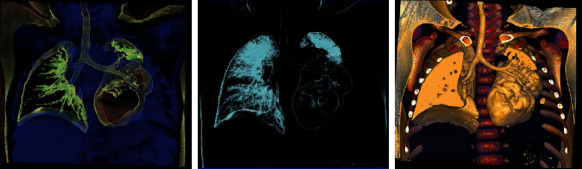
Noncontrast frontal view chest CT, image reformatting volume rendered display: coronal reformation in standard lung window to allow three-dimensional view. Pneumatoceles, thin-walled cavities developing secondary to necrosis.

**Figure 8:**
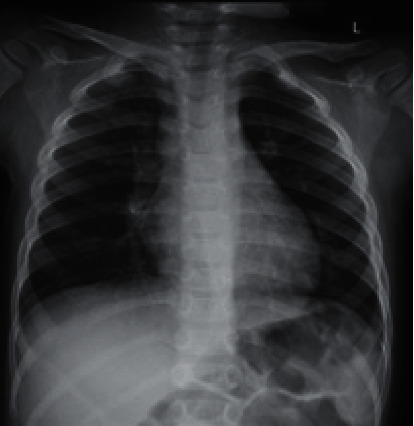
Follow-up chest radiograph obtained 6 months after hospitalization. Frontal CXR has no apparent abnormality.

On the first day at home, she had worsening symptoms of fever and shortness of breath. Her parents took her back to the ED. Upon arrival to the ED, her body temperature was 38.7^o^C, respiratory rate was 52 times per minute, and heart rate was 180 beats per minute. She was admitted to hospital again, and laboratory tests showed elevated CRP of 96 mg/l and high white blood cell count of 21,400 *μ*l^−1^. Her laboratory result comparison is shown in Table 1. A second CXR (Figure 2) was obtained in the radiology department. AP and lateral view demonstrated focal area of consolidation dominant in the left inferior lobe. On frontal view, there are multiple small lucency in the left lung field suspicious for possible pneumonia with air cavitation or suspected combined with congenital pulmonary airway malformation (CPAM). Compared to the previous chest X-ray (Figure 1), no lucency was seen, possibly pneumonia with air cavitation. During the hospitalization, the condition became worse, and on day 8, she was followed by a follow-up by CXR (Figure 3) combined with lung ultrasonography (LUS) (Figures 4 and 5), showed multiple large air-filled cavitary lesions in the left lung. Pleural effusion (transudative), pleural thickening, heterogeneous parenchymal echo texture, and no pneumothorax was visible. On the same day, chest CT scan was done without contrast, as the parent refused for contrast administration (Figures 5–7). CT scan images revealed consolidation with multiple cavities or pneumatoceles consistent with necrotizing pneumonia, left pleural effusion density was <25 Hounsfield units (HU), and collapse of partial left superior lobe. The blood culture was sterile, cytology analysis did not reveal any malignant tumor cells, and fast acid bacilli stain and GeneXpert also showed negative results. The patient's condition was getting better with aggressive intravenous antibiotic therapy with meropenem, amikacin, and azythromycin. On the third day of this hospitalization period, the patient demonstrated significant clinical improvement, including resolution of fever and respiratory distress. Seven days later, she was discharged and advised to continue oral antibiotic therapy. She was feeling well and was back to her usual activities without any respiratory symptoms. Follow-up chest radiograph was obtained 6 months after hospitalization and revealed no abnormalities Figure 8).

## 3. Discussion

Necrotizing pneumonia (NP) refers to the development of necrosis, liquefaction, and cavitation of the lung parenchyma by infectious pathogens. It is said that approximately 4% of community pneumonia are necrotizing, although this percentage seems to increase [3]. Lack of pulmonary and bronchial vascular supply may result devitalization of lung parenchyma. The lack of blood supply to the underperfused areas may result in reduced delivery of antibiotics, allowing for uncontrolled infection and further destruction of lung tissue [4].

Necrotizing pneumonia usually develops over a few days and presents acutely with severe sepsis. Low white blood cell count, bloody cough, and hyperpyrexia were found with imaging evidence of tissue necrosis and microabscesses. Pulmonary gangrene is often present; large cavities are formed when small abscesses join together. In contrast with NP, pulmonary abscesses tend to appear chronically with a long history of fever and night sweats [5]. In this case, the child develops acute symptoms which occurred within days.

Radiology plays a major role in both the diagnosis and treatment of complications of pneumonia in children. The imaging modality of choice is plain chest radiography, including for community-acquired pneumonia (CAP). Computed tomography (CT) on the other hand is not a first-line imaging tool for children with suspected uncomplicated CAP. It is considered when complications are suspected or where there is difficulty in differentiating CAP from other diseases [6]. Lung ultrasonography (LUS) is another modality of choice with a feasible, portable, and nonionizing radiation technique that has been used to help diagnosing pulmonary pathologies in children. Compared with X-ray and CT, LUS has higher sensitivity and specificity in the diagnosis of CAP in pediatric patients [7].

A multidisciplinary team of pediatric respiratory physicians, pediatric or thoracic surgeons, and infectious diseases consult is often required in the case of NP. The objectives are to control and ultimately reverse the pathobiologic changes associated with NP. These include providing supplemental oxygen to relieve hypoxia, adequate pain management to reduce pleuritic pain caused by the infection and improve ventilation, administering long course of antibiotic therapy, and decreasing any mass effect or intrathoracic pressure by draining gas and/or intrapleural fluid [8]. Conservative therapy relies on intravenous (IV) antibiotics. The recommended first-line therapy for severe CAP without complication in children is IV penicillin or ampicillin and then will need to include antistaphylococcal antibiotics, such as oxacillin or flucloxacillin. When coinfection with *M. pneumoniae* is suspected, a macrolide such as IV clarithromycin or azithromycin is added [1]. Other than conservative therapy, some authors recommend that invasive procedures be performed only if the child remains seriously ill and recurrent despite appropriate antibiotic therapy [9]. In this case, we give conservative therapy to the patient with aggressive intravenous antibiotic therapy with meropenem, amikacin, and azithromycin, and the result was remarkable.

## 4. Conclusion

Necrotizing pneumonia represents a rare but serious complication of pneumonia in children. It should be suspected in pneumonia with progressive clinical deterioration, high fever, and respiratory distress despite adequate antibiotic therapy at least 72 hours. LUS and CT represent well-established modalities for evaluation of complicated pediatric pneumonia. CT scan should not be delayed to confirm diagnosis and provide treatment. Initially, conservative treatment with supplemental oxygen and IV antibiotics should be administered, while surgical and invasive treatment should be performed if the child remains seriously ill and the infection is refractory.

## Figures and Tables

**Table 1:** Comparison of laboratory values between the first and second hospitalization period.

Parameter (normal values)	1^st^ hospitalization	2^nd^ hospitalization
Hemoglobin (10.8–12.8 g·dL^−1^)	10.2	11.6
WBC count (5,000–21,000 *μ*^−1^)	10,100	21,400
Platelet count (150,000–450,000 *μ*l^−1^)	420,000	702,000
Hematocrit (35–43%)	32.0	37.0

Differential count		
Basophil	0	0
Eosinophil	0	0
Band neutrophil	4.0	1.0
Segmented neutrophil	50.0	81.0
Lymphocyte	45.0	17.0
Monocyte	1.0	1.3
C-reactive protein (<2 mg · L^−1^)	24	96
